# RETRACTED: Teklemariam et al. Isolation and Characterization of a Novel Lytic Phage, vB_PseuP-SA22, and Its Efficacy against Carbapenem-Resistant *Pseudomonas aeruginosa*. *Antibiotics* 2023, *12*, 497

**DOI:** 10.3390/antibiotics13090814

**Published:** 2024-08-28

**Authors:** Addisu D. Teklemariam, Rashad R. Al-Hindi, Mona G. Alharbi, Ibrahim Alotibi, Sheren A. Azhari, Ishtiaq Qadri, Turki Alamri, Ahmed Esmael, Steve Harakeh

**Affiliations:** 1 Department of Biological Sciences, Faculty of Science, King Abdulaziz University, Jeddah 21589, Saudi Arabia; ademeke16@yahoo.com (A.D.T.); mgalharbi@kau.edu.sa (M.G.A.); sazhari@kau.edu.sa (S.A.A.); ishtiaq80262@yahoo.com (I.Q.); 2 Health Information Technology Department, Applied College, King Abdulaziz University, Jeddah 21589, Saudi Arabia; ialotibi@kau.edu.sa; 3 Family and Community Medicine Department, Faculty of Medicine in Rabigh, King Abdulaziz University, Jeddah 21589, Saudi Arabia; olbalamri7@kau.edu.sa; 4 Botany and Microbiology Department, Faculty of Science, Benha University, Benha 13518, Egypt; 5 Nebraska Center for Virology, University of Nebraska-Lincoln, Lincoln, NE 68583, USA; 6 King Fahd Medical Research Center, Yousef Abdullatif Jameel Chair of Prophetic Medicine Application, Faculty of Medicine, King Abdulaziz University, Jeddah 21589, Saudi Arabia

The journal retracts the article, “Isolation and Characterization of a Novel Lytic Phage, vB_PseuP-SA22, and Its Efficacy against Carbapenem-Resistant *Pseudomonas aeruginosa*”, cited above.

Following publication, concerns were brought to the attention of the publisher regarding an overlap of the images.

Adhering to our complaints procedure, an investigation was conducted that confirmed the overlap of Figure 8C with a figure in a previously published article [1], listing different experimental conditions. While the authors fully cooperated with the Editorial Office during the investigation, they were unable to satisfactorily explain the overlap of the figures or meet the required quality standards for raw images in order to consider a correction as per the journal’s original image requirements policy (https://www.mdpi.com/journal/antibiotics/instructions#oriimages). As a result, the Editorial Board and Editor-in-Chief have lost confidence in the validity of the findings and decided to retract this paper as per MDPI’s retraction policy (https://www.mdpi.com/ethics#_bookmark30).

This retraction was approved by the Editor-in-Chief of the journal *Antibiotics*.

The authors have agreed to this retraction.

## References

[1] Teklemariam A.D., Al-Hindi R.R., Alharbi M.G., Alotibi I., Azhari S.A., Qadri I., Alamri T., Esmael A., Harakeh S. (2023). RETRACTED: Isolation and Characterization of a Novel Lytic Phage, vB_PseuP-SA22, and Its Efficacy against Carbapenem-Resistant *Pseudomonas aeruginosa*. Antibiotics.

